# Investigating the applicability domain of the hiPSC-based PluriLum assay: an embryotoxicity assessment of chemicals and drugs

**DOI:** 10.1007/s00204-023-03675-1

**Published:** 2024-02-04

**Authors:** Andreas Frederik Treschow, Maria João Valente, Karin Lauschke, Bjørn Holst, Anders Reenberg Andersen, Anne Marie Vinggaard

**Affiliations:** 1 Cell Toxicology Team, National Food Institute, Technical University of Denmark, Kongens Lyngby, Denmark; 2 Bioneer A/S, Hørsholm, Denmark; 3 Department of Applied Mathematics and Computer Science, Technical University of Denmark, Kongens Lyngby, Denmark; 4 Present Address: Cell Therapy TRU, Novo Nordisk A/S, Måløv, Denmark

**Keywords:** Developmental toxicity, Embryotoxicity, hiPSCs, Embryoid bodies, PluriLum, Cardiomyocyte differentiation

## Abstract

To meet the growing demand for developmental toxicity assessment of chemicals, New Approach Methodologies (NAMs) are needed. Previously, we developed two 3D in vitro assays based on human-induced pluripotent stem cells (hiPSC) and cardiomyocyte differentiation: the PluriBeat assay, based on assessment of beating differentiated embryoid bodies, and the PluriLum assay, a reporter gene assay based on the expression of the early cardiac marker *NKX2.5*; both promising assays for predicting embryotoxic effects of chemicals and drugs. In this work, we aimed to further describe the predictive power of the PluriLum assay and compare its sensitivity with PluriBeat and similar human stem cell-based assays developed by others. For this purpose, we assessed the toxicity of a panel of ten chemicals from different chemical classes, consisting of the known developmental toxicants 5-fluorouracil, all-*trans* retinoic acid and valproic acid, as well as the negative control compounds ascorbic acid and folic acid. In addition, the fungicides epoxiconazole and prochloraz, and three perfluoroalkyl substances (PFAS), PFOS, PFOA and GenX were tested. Generally, the PluriLum assay displayed higher sensitivity when compared to the PluriBeat assay. For several compounds the luminescence readout of the PluriLum assay showed effects not detected by the PluriBeat assay, including two PFAS compounds and the two fungicides. Overall, we find that the PluriLum assay has the potential to provide a fast and objective detection of developmental toxicants and has a level of sensitivity that is comparable to or higher than other in vitro assays also based on human stem cells and cardiomyocyte differentiation for assessment of developmental toxicity.

## Introduction

Toxicological assessment of adverse effects from chemicals during embryonic development is key to protecting future generations from exposure to harmful substances in utero (Worley et al. 2018)*.*

Currently, tests for developmental toxicity mainly rely on animal models. Although essential, in vivo toxicity testing comes with challenges in predicting human responses and several ethical issues related to the suffering of animals, besides being low-throughput, costly, and labor intensive. The current golden standard for developmental toxicity testing/embryotoxicity testing accepted by regulatory bodies is described in the OECD test guideline 414 (Luconi et al. 2022; Piersma et al. 2022). This guideline describes how rodents, predominantly rats and rabbits are exposed to chemicals throughout a full gestation period whereafter the effects on embryonic/fetal development are assessed upon killing of the animal one day before delivery (OECD 2018). Since the European Chemicals Agency adopted the Registration, Evaluation, Authorization and Restriction of Chemicals act in 2007, which aimed to test the toxicity of 68,000 industrial chemicals, approximately 1.3 million animals have been used to investigate for developmental toxicity according to test guideline 414 (Knight et al. 2023; Rovida et al. 2023).

One of the main issues with this approach is that the use of animal data to infer toxicity in humans suffers from the inherent problem of species extrapolation (Olson et al. 2000; Daston and Knudsen 2010). The most famous incident of this interspecies variation of adverse effects is the thalidomide scandal, where the absence of toxicity in rats led to the false assumption of safety of the drug in humans. Thalidomide was prescribed to pregnant women as a treatment for morning sickness resulting in tragic limb malformation in children and an increased rate of miscarriages (Vargesson 2015).

In accordance with the principles of the 3Rs, i.e. replace, reduce and refine (Russell and Burch 1960), it is envisioned that risk assessment of chemicals in the future will rely increasingly on data from non-animal sources and therefore development of New Approach Methodologies (NAMs) with relevant predictive value for humans is key for the progression of this field within toxicology (European Chemicals Agency 2016; USEPA 2021; Escher et al. 2022; Piersma et al. 2022).

Based on this, our team has previously developed the human-induced pluripotent stem cells (hiPSC) developmental toxicity PluriBeat assay (Lauschke et al. 2020). By generating 3D embryoid bodies (EBs) that display similarities to the blastocyst (Brickman and Serup 2017; Zeevaert et al. 2020), and differentiating them towards a cardiac fate, we can test for effects by measuring reduction in beating of the cardiospheres following chemical exposure during the 7-day period of differentiation (Lauschke et al. 2020). The heart is the first functional organ in the developing embryo with onset of beating on the 21st day after fertilization, and the assay is designed to act as a surrogate for early embryonic development (Lauschke et al. 2020).

The invention of hiPSCs, generated by cellular reprogramming of adult somatic cells (Takahashi and Yamanaka 2006), has made human stem cells available without the ethical implications related to human embryonic stem cells (hESCs) (Moradi et al. 2019). Comparison between the predictive value of hiPSCs and hESCs have shown hiPSCs as a viable alternative to hESCs in developmental toxicity testing through investigations of chemical exposure to *e.g.* valproic acid (VPA) (Shinde et al. 2016) and all-*trans* retinoic acid (atRA) (Mayshar et al. 2011).

Using the PluriBeat assay, we have previously detected developmental toxicity of the teratogen thalidomide, the fungicide epoxiconazole (Lauschke et al. 2020), and of the three perfluoroalkyl substances (PFAS), perfluorooctanoic acid (PFOA), perfluorooctanesulfonic acid (PFOS), and undecafluoro-2-methyl-3-oxahexanoic acid (GenX) (Davidsen et al. 2021).

To improve the assay and to eliminate the subjective visual assessment of the EB contractions, a reporter gene version of the assay was established (Lauschke et al. 2021b). The gene *NKX2.5* was chosen as it is a key gene coding for homeobox transcription factor expressed during early cardiac differentiation (Zhang et al. 2015; Lauschke et al. 2020). Using CRISPR/Cas9, we fused the gene *NKX2.5* with a T2A-*Nluc* construct coding for co-expression of an artificial luciferase (England et al. 2016; Lauschke et al. 2021b)*.* This resulted in the hiPSC-based luciferase reporter gene assay named the PluriLum assay.

We have previously shown that the PluriLum assay can detect the effects of thalidomide at lower concentrations than the PluriBeat, paving the way for a faster and unbiased data-acquisition with a more sensitive readout (Lauschke et al. 2021b).

In this work, we aimed at further characterizing the applicability domain and the sensitivity of the PluriLum assay. We have screened a panel of ten chemical compounds covering both positive controls (5-Fluorouracil (5-FU), atRA and VPA), negative controls (ascorbic acid and folic acid), three PFAS (PFOS, PFOA and GenX) and two fungicides (epoxiconazole and prochloraz) for developmental toxicity effects using the PluriLum assay and compared to data from the PluriBeat assay and the literature.

## Materials and methods

### Reagents and chemicals

Corning® Matrigel® hESC-Qualified Matrix and Corning® ITS Premix Universal Culture Supplement were obtained from Corning Inc (NY, USA). mTeSR™1 medium was purchased from STEMCELL Technologies Inc. (Vancouver, Canada). TrypLE™, Penicillin–Streptomycin-Glutamine (PSG), KnockOut™ DMEM medium, human fibroblast growth factor-basic (FGF2), activin A, as well as 60 mm cell culture dishes and 96-well Polystyrene Conical Bottom MicroWell™ plates, were supplied by Thermo Fisher Scientific Inc. (Massachusetts, EUA). L-Ascorbic acid 2-phosphate trisodium salt (Asc), sodium selenite, and human transferrin were purchased from Merck KGaA (Darmstadt, Germany). Rho kinase inhibitor and 6-(2-(4-(2,4-Dichlorophenyl)-5-(4-methyl-1H-imidazol-2-yl)-pyrimidin-2-ylamino)ethyl-amino)-nicotinonitrile (CHIR99021) were purchased from Abcam Plc (Cambridge, UK) and Axon Medchem (Groningen, the Netherlands), respectively. Human bone morphogenetic protein 4 (BMP4) and 4-(2-Methyl-4-pyridinyl)-N-[4-(3-pyridinyl)phenyl]benzeneacetamide (Wnt-C59) were obtained from Bio-Techne (Minnesota, USA). The test compounds prochloraz (CAS number 67747–09-5), epoxiconazole (CAS number 133855–98-8), ascorbic acid (CAS number 50–81-7), folic acid (CAS number 59–30-3), atRA (CAS number 302–79-4), PFOA (CAS number 335–67-1), PFOS (CAS number 2795–39-3), 5-FU (CAS number 51–21-8) and VPA sodium salt (CAS number 1069–66-5) were obtained from Merck KGaA (Darmstadt, Germany). GenX (CAS number 13252–13-6) was supplied by SynQuest Laboratories, Inc. (Florida, USA). All compounds were of analytical grade, with assessed purity ≥ 97%.

### Cell culture maintenance

The parental hiPSC cell line, BIONi010-C, was established at Bioneer A/S (Bioneer A/S, Hørsholm, Denmark), from normal adult human dermal fibroblasts (Rasmussen et al. 2014). The induced pluripotent stem cell line is available via the European Bank for stem cells (https://ebisc.org). The genetically modified version of this cell line, *NKX2.5*-T2A-*Nluc*-44.37, used in this work was established and quality assessed in collaboration with Bioneer A/S (Lauschke et al. 2021b). The hiPSCs culture was maintained on Matrigel®-coated cell culture dishes in mTeSR™1 medium. Cells were incubated at 37 °C and 5% CO_2_, in a humid environment. Culture medium was changed every day and cultures were split approximately once a week using 0.02% EDTA in DPBS. Cells were kept in culture through passages 33–47.

### Cardiomyocyte differentiation

The hiPSCs were differentiated into cardiomyocytes as described in previously published work by the group (Lauschke et al. 2020). The protocol consists of an 7-day cardiomyocyte differentiation protocol. Passages no. 37–47 were used for differentiation.

Near confluent hiPSC cultures were dissociated into single cells by incubation with TrypLE™ for 4 min at 37 °C. Cells were then resuspended in mTeSR-ROCK medium [mTeSR™1 medium containing 10 µM Rho kinase inhibitor and 1% (V/V) PSG]. 5,000 single cells/well were then seeded into 96-well Polystyrene Conical Bottom MicroWell™ plates, spun down at 500*g* for 5 min, and incubated overnight at 37 °C and 5% CO_2_. After a 19-h overnight incubation, EBs had been formed in the bottom of the conical wells.

The medium was exchanged after 19 h into D0 differentiation medium [KnockOut™ DMEM medium containing 10 µM Rho kinase inhibitor, 1% (V/V) PSG, 0.1% (V/V) ITS, 10 ng/mL FGF2, 10 ng/mL activin A, 2.5 µM CHIR99021 and 1 ng/mL BMP4]. Medium was changed on D1, D2, D3 into TS-medium [KnockOut™ DMEM medium containing 1% (V/V) PSG, 40 nM sodium selenite, 5.5 µg/mL human transferrin and 25 µM Asc], Wnt-medium [TS-medium containing 400 nM Wnt-C59] and TS-medium, respectively. On D6, medium was replaced with fresh TS-medium. The differentiation was assessed after additional 24 h, i.e. on D7.

### Chemical exposures

Test compounds were prepared in stock solutions concentrated by a factor of 1000 in relation to the highest final exposure concentration. The following compounds were prepared in DMSO stock-solutions: prochloraz (10 mM), epoxiconazole (20 mM), 5-FU (3.2 mM), folic acid (100 mM), atRA (200 µM), PFOA (50 mM) and PFOS (50 mM). VPA (300 mM) was prepared in 96% ethanol. Ascorbic acid (500 mM) and GenX (100 mM) were prepared MilliQ water. For exposure experiments, the dilutions of chemicals were added in a ratio of 1:1000 to the respective media on the experimental days: D1, D2, D3 and D6 allowing for a constant vehicle (v/v) concentration of 0.1% in all wells. For each individual experimental condition, 20–32 EBs were exposed.

### Scoring of embryoid body contraction

The contractility of each individual EB was assessed in the PluriBeat assay by visually evaluating the beating on D7 using a light microscope (Nikon Eclipse Ts2, Tokyo, Japan). Each EB was assessed for up to 15 s. The level of contractility was given a score using the following criteria: score = Full Beat, if the whole EB was contracting; score = Partial Beat, if the EB or smaller areas were partially contracting; score = No Beat, if the EB showed no visible movement. For each experimental condition, the scoring outcome was used as a surrogate to assess the developmental effect of the chemical exposure compared to vehicle controls. A prerequisite for a successful experiment was that > 90% of all vehicle controls within each plate should be fully beating on D7.

### Analysis of *NKX2.5* activation by luminescence measurements

*NKX2.5* activation in the PluriLum assay was analyzed by luminescence measurements. After EBs had undergone beat scoring, the luminescence of the individual EBs was measured using the Promega Nano-Glo® Luciferase Assay System (Promega, Wisconsin, USA), according to the manufacturer’s protocol. The EBs were transferred from the microtiter plate in a volume of 40 µL medium into flat bottomed white 96-well plates for luminescence measurements. 40 µL of Nano-Glo® Luciferase Assay Substrate was added to each well and pipetted up and down 10 times for complete dissociation of the EBs. Measurements were performed on a PerkinElmer EnSpire 2300 Multimode Microplate Reader (PerkinElmer, Inc., Massachusetts, USA).

### Data processing and statistical analysis

Statistical analysis on luminescence data was performed using GraphPad Prism 9 (version 9.4.1). All experiments were performed in three independent experiments. Each experimental condition contained 20–32 technical replicates (EBs).

Luminescence data points were resected according to the following criteria: values from wells without EBs were removed (luminescence value < 50,000). Luminescence data for each EB were normalized to the average of the experimental vehicle controls. The value for each experimental condition is the average of the normalized RLU of the EBs. Results are presented as mean ± standard deviation. Statistical analysis was performed using one-way ANOVA without matching, followed by multiple comparisons using the Bonferroni post-hoc test.

The statistical analysis of the beat score data was performed in R. Beat scores were treated as ordinal categories in a Proportional Odds Logistic Regression (POLR). Specifically, we fitted a POLR model, $Y=\beta x-{\theta }_{i}$, for each concentration, where $x$ is the concentration of the compound, $\beta$ is the slope, and ${\theta }_{i}$ the intercept associated with beat scores larger than $i$. Thus, $i$ can take on values of 0 and 1, respectively. The response, $Y$, is the cumulated log-odds of obtaining a beat score larger than $i$. Each model contained the data for the control and the respective concentration. We tested the null-hypothesis that the concentration had no effect on the beat score by conducting a likelihood-ratio test comparing the model to the corresponding model without slope, i.e. $Y=-{\theta }_{i}$.

We fitted the parameters for the POLR models using the ‘polr’ function from the ‘MASS’ package in R.^1^ The likelihood-ratio tests were conducted by assuming the difference between the deviances of the models followed a chi-square distribution with 1 degree of freedom under the null-hypothesis. The *p*-values were calculated from the right tail of the distribution using the function ‘pchisq’ in R. *P*-values lower than 0.05 were considered statistically significant.

## Results

### Effect of 5-fluorouracil and all-trans retinoic acid on cardiomyocyte differentiation

**Fig. 1:**
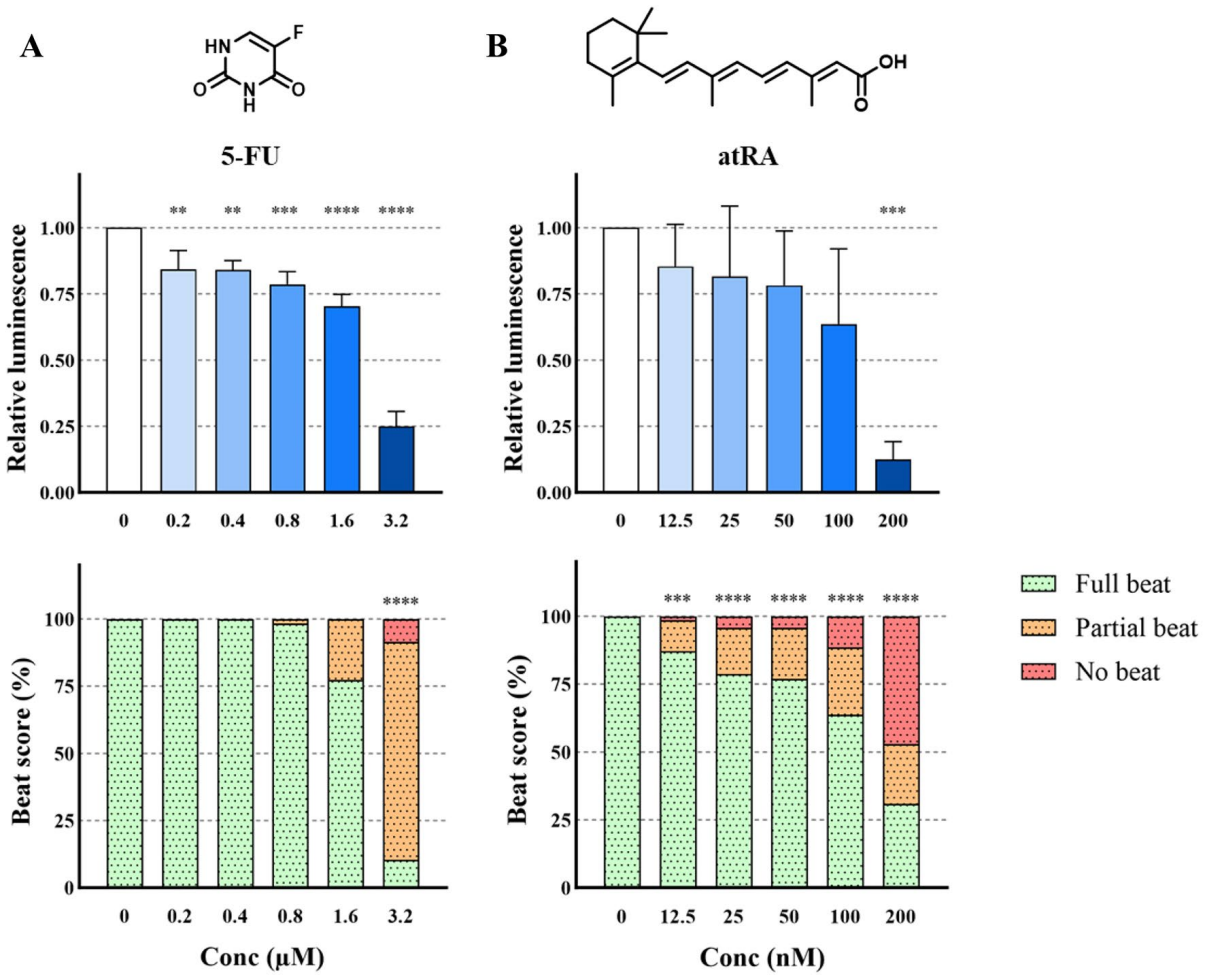
Chemical structure and effect on luminescent output (relative to control) in the PluriLum assay and on beat score in the PluriBeat assay of 5-fluorouracil (5-FU; **A**), and all-*trans* retinoic acid (atRA, **B**). **p < 0.01, ***p < 0.001, ****p < 0.0001 *versus* control

As depicted in Fig. 1A, the decline of luminescence intensity after exposure to 5-FU was significant from the lowest concentration tested, i.e. 0.2 µM (*p* < 0.01). The effect was concentration-dependent, with around 75% reduction at 3.2 µM (*p* < 0.0001). At the same time, reduction in the number of beating EBs was significant only at the highest exposure concentration 3.2 µM.

The exposure to atRA (Fig. 1B) significantly affected the luminescence intensity at the highest exposure concentration of 200 nM (*p* < 0.001), whereas beat score was significantly reduced following atRA exposure from the lowest concentration of 12.5 nM, with an increasing effect throughout the exposure range up to 200 nM (*p* < 0.001).

### Effects of valproic acid, folic acid, and ascorbic acid on cardiomyocyte differentiation

**Fig. 2:**
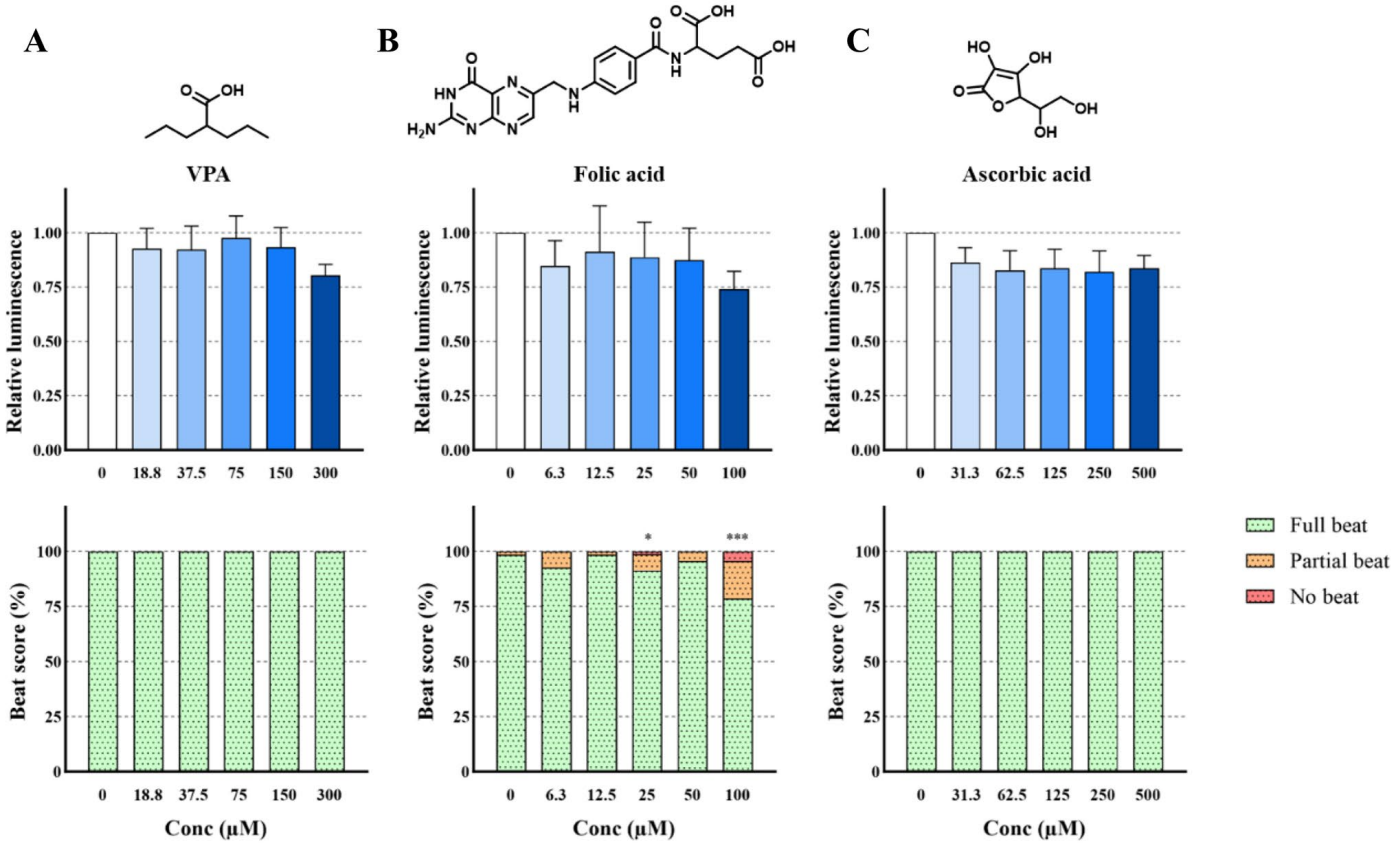
Chemical structure and effect on luminescent output (relative to control) in the PluriLum assay and on beat score in the PluriBeat assay of valproic acid (VPA, **A**), folic acid (**B**), and ascorbic acid (**C**). *p < 0.05, ***p < 0.001 *versus* control

No significant effect was elicited by exposure to VPA in either readout (Fig. 2A). There were also no observed significant effects of exposure to folic acid using luminescence reduction as an endpoint. There was, however, a significant reduction in beating in response to concentrations of 25 and 100 µM folic acid (Fig. 2B; *p* < 0.05). Similarly to VPA, exposure to ascorbic acid (Fig. 2C) had no effect neither on the beating of the EBs, nor on the luminescence intensity.

### Effect of perfluoroalkyl substances on cardiomyocyte differentiation

**Fig. 3:**
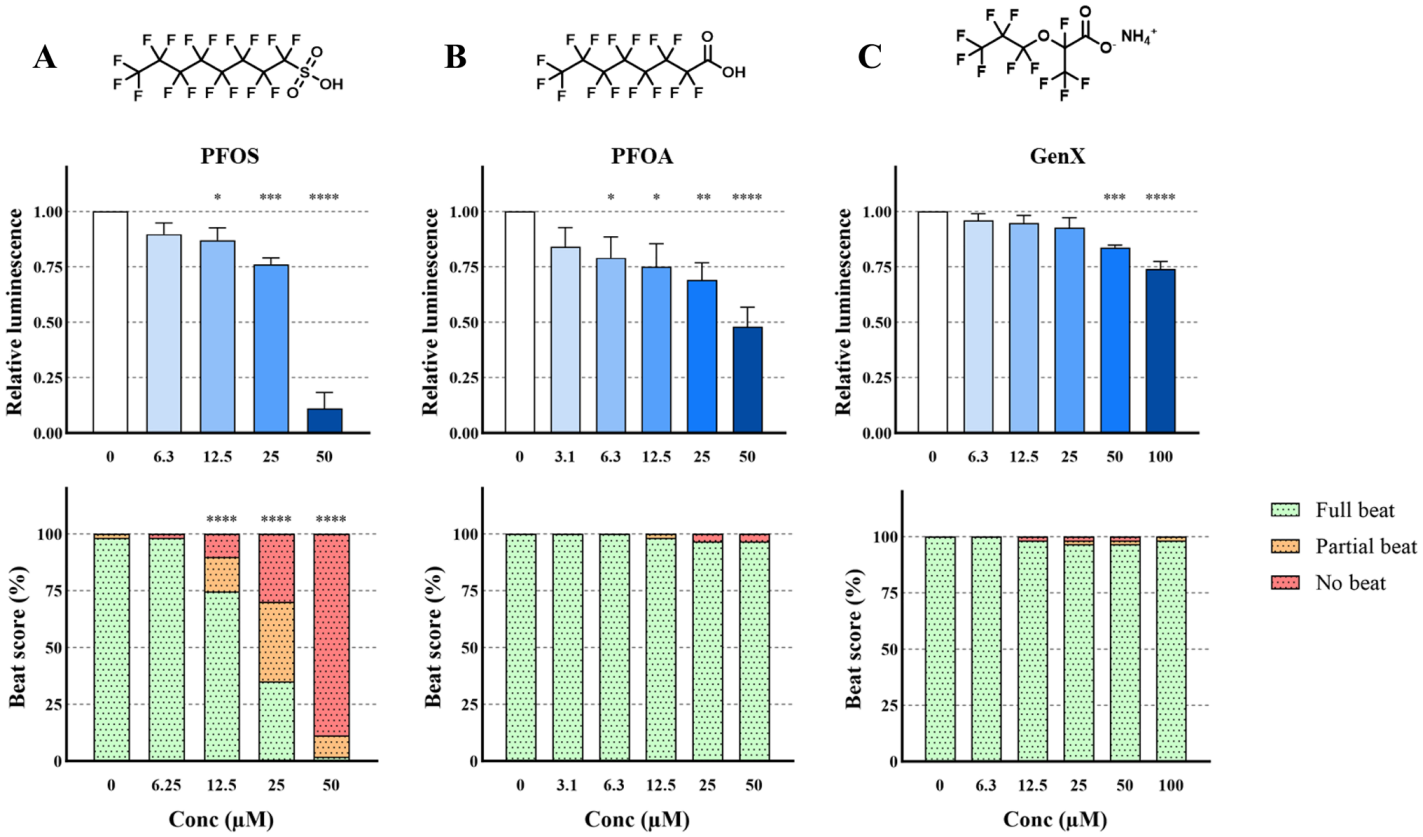
Chemical structure and effect on luminescent output (relative to control) in the PluriLum assay and beat score in the PluriBeat assay of perfluorooctanesulfonic acid (PFOS; **A**), perfluorooctanoic acid (PFOA; **B**), and undecafluoro-2-methyl-3-oxahexanoic acid (GenX; **C**). *p < 0.05, **p < 0.01, ***p < 0.001, ****p < 0.0001 *versus* control

The three perfluorinated compounds PFOA, PFOS, and GenX all affected cardiomyocyte differentiation but with different outcomes (Fig. 3). Exposure to PFOS resulted in a decrease of beating EBs from a concentration of 12.5 µM (*p* < 0.0001). At the same exposure level, the luminescence output was also negatively affected (*p* < 0.05). Exposure to 50 µM PFOS resulted in almost no fully contracting EBs, while the luminescence output was reduced tenfold compared to control (*p* < 0.0001). The effect levels were comparable between the two readouts for exposure to PFOS (Fig. 3A).

Exposure to PFOA did not show significant effects on the beating of the cardiomyocytes, while luminescence was affected significantly (Fig. 3B). The luminescence response was reduced at exposure concentrations from 6.3 µM PFOA (*p* < 0.05). This tendency increased to a 50% reduction in luminescence signal when exposed to 50 µM PFOA (*p* < 0.0001) (Fig. 3B).

Like PFOA, exposure to GenX had no impact on the beat score, whereas a significant reduction in luminescence was observed at exposure concentrations of 50 and 100 µM (Fig. 3C; *p* < 0.001).

### Effect of conazole fungicides on cardiomyocyte differentiation

**Fig. 4:**
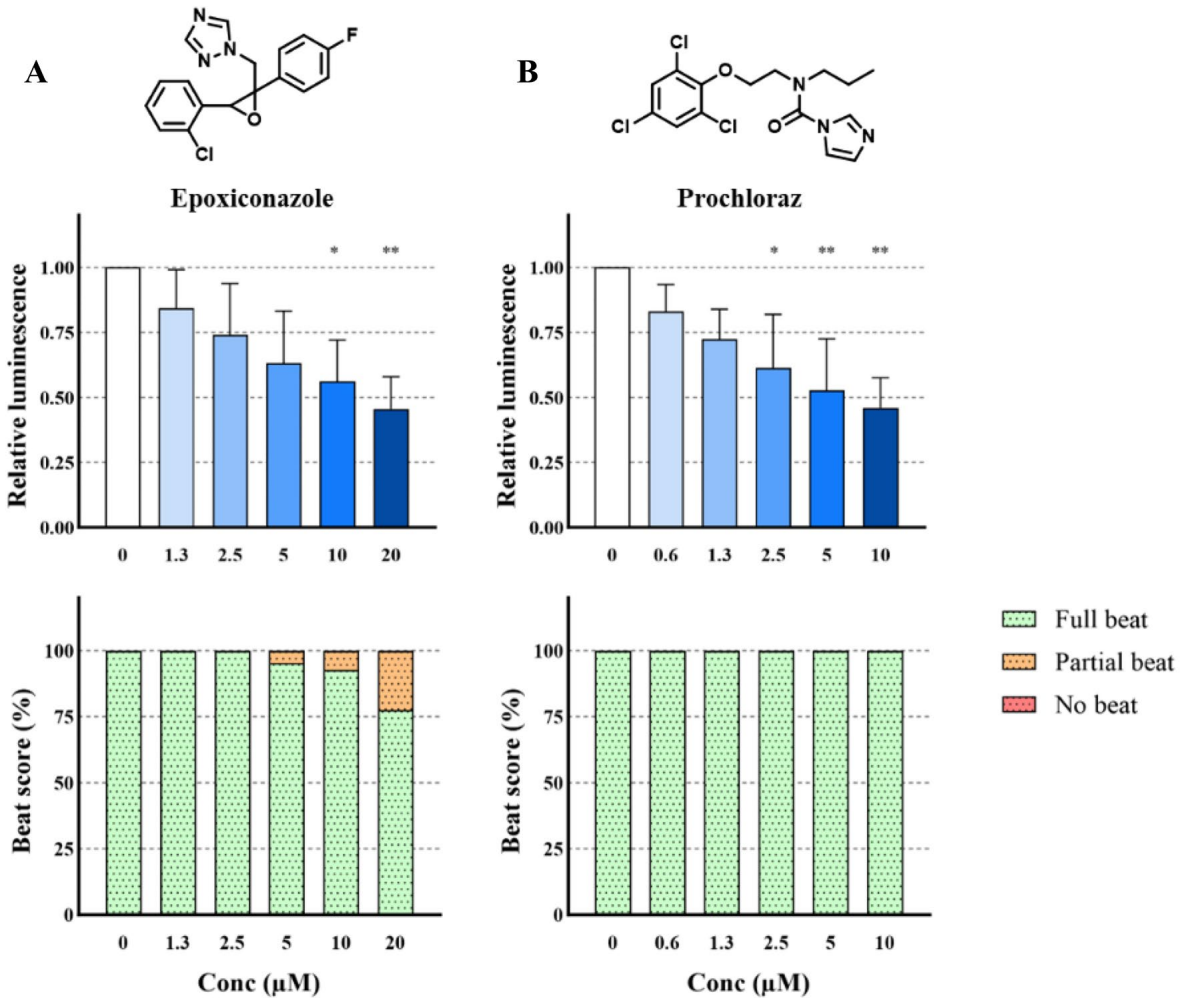
Chemical structure and effect on luminescent output (relative to control) in the PluriLum assay and on beat score in the PluriBeat assay of epoxiconazole (**A**) and prochloraz (**B**). *p < 0.05, **p < 0.01 *versus* control

No significant effect on beating was observed after exposure to prochloraz or epoxiconazole during differentiation at any of the test concentrations (Fig. 4).

On the other hand, significant reductions in the luminescence intensity were observed from exposure to 10 and 20 µM epoxiconazole (*p* < 0.05). For prochloraz, the luminescence intensity was significantly reduced by exposures from 2.5 µM and up to 10 µM (*p* < 0.05).

## Discussion

In this study, we investigated the applicability domain of the PluriLum assay and related our findings to current knowledge on developmental toxicity of the test compounds. In addition, we have compared our findings and the performance of the assay to similar in vitro test systems designed to utilize embryonic or pluripotent stem cells and cardiomyocyte differentiation for assessment of developmental toxicity.

Since the creation of the mouse embryonic stem cell test (mEST), cardiomyocyte differentiation has been used as an endpoint for developmental toxicity testing. However, the use of this differentiation fate in human stem cells is still limited. We have performed a literature search on hESC and hiPSC-based models that rely on cardiomyocyte differentiation for developmental toxicity testing. A summary of a total of 23 published studies is presented in (Table 1).

**Table 1:** List of published developmental toxicity studies based on cardiomyocyte differentiation in human stem cells

Cell type	Cell line	Protocol	Endpoints/methods	Tested substances	References
hiPSCs					
3D	WTC Yale-WT	Organoids formed in micropatterned well stencils 24-day protocol	Cardiomyocyte contraction Calcium flux Morphology assessment RT-qPCR Confocal imaging Flow cytometry	Thalidomide, 13-*cis* retinoic acid, atRA, doxycycline, phenytoin, lithium carbonate, rifampicin, amoxicillin, doxylamine succinate	Hoang et al. (2021)
	BIONi010-C IMR90-1 IMR90-4	EBs generated in V-shaped plates 8-day protocol	Cardiomyocyte contraction EBs size assessment RT-qPCR	PFOS, PFOA, GenX	Davidsen et al. (2021)
	BIONi010-C	EBs generated in V-shaped plates 8-day protocol	RNAseq RT-qPCR	Epoxiconazole	Lauschke et al. (2021a, b)
	BIONi010-C BIONi010-C-*NKX2.5-T2A-Nluc*	EBs generated in V-shaped plates 8-day protocol	*NKX2.5*-reporter quantification Cardiomyocyte contraction RT-qPCR	Thalidomide, VPA	Lauschke et al. (2021a, b)
	BIONi010-C IMR90-1 IMR90-4	EBs generated in V-shaped plates 8-day protocol	Cardiomyocyte contraction RT-qPCR	Thalidomide, VPA, epoxiconazole	Lauschke et al. (2020)
	Created by authors	EBs generated in Aggrewell 8-day protocol + FBS	Cardiomyocyte contraction	VPA, thalidomide, ascorbic acid, aminopterin, methotrexate, atRA, tetracycline, lithium, carbonate, VPA, phenytoin, 5-FU, warfarin, isoniazid, saccharin, penicillin G	Aikawa et al. (2014; Aikawa 2020)
2D	Gibco™ episomal line	24-well plates 14-day protocol	Cardiomyocyte contraction Immunostaining RT-qPCR	Thalidomide, VPA, fingolimod, diltiazem, warfarin, hydroxyurea, thiamine, folic acid, amoxicillin, vildagliptin, metoclopramide, progesterone, 1,2-propylene glycol, saccharin, sitagliptin, methylmercury, hydrochlorothiazide, pazopanib, imatinib, tacrolimus	Jamalpoor et al. (2022)
	iPS11	48-well plate 10-day protocol	Cardiomyocyte contraction RT-qPCR Immunostaining Flow cytometry	Penicillin G, 5-FU	Galanjuk et al. (2022)
	RIV9	Culture plate 25-day protocol + FBS	Cardiomyocyte contraction RT-qPCR	atRA, penicillin G, 5-FU, cigarettes extracts (mixtures), Snus tobacco extracts (mixtures)	Walker et al. (2021)
	unknown	Culture plate 10-day protocol	Cardiomyocyte contraction RT-qPCR	Ribavirin	Ye et al. (2020)
	unknown	Culture plate 10-day protocol	RT-qPCR Proliferation assay during differentiation	Arsenic trioxide	Bao et al. (2019)
hiPSCs and hESCs					
2D	hiPSCs: C-15, hESCs: H1	12 well plate 10-day protocol, with intermediate assessments (D0, D2, D6)	RNAseq ATAC-seq Flow cytometry RT-qPCR Western blotting Immunostaining	13-*cis* retinoic acid	Liu et al. (2018)
hESCs					
3D	H9-TNNT2^tm1^ (GFP)Bcgen	EBs generated in Aggrewell 10-day protocol + FBS	High content analysis of *TNNT2*-GFP reporter quantification	Zearalenone, 5-fluorouracil, diphenylhydantoin, penicillin G	Cao et al. (2019)
	H9	EBs generated in Aggrewell 10-day protocol + FBS	RT-qPCR	Deoxynivalenol, 5-FU, penicillin G	Fang et al. (2018)
	H9	EBs generated in low attachment plate 24-day protocol	Cardiomyocyte contraction RT-qPCR	Trichloroethylene	Jiang et al. (2016)
	PKU1.1	EBs generated in low attachment plate 28-day protocol FBS +	Cardiomyocyte contraction RT-qPCR	Cyclophosphamide	Zhu et al. (2011)
3D/2D	NKX2.5^eGFP/w^	3D: EBs generated in ultra-low attachment plate 10-day protocol 2D: 96-well plate 10-day protocol	Cardiomyocyte contraction *NKX2.5*-eGFP reporter quantification RT-qPCR Immunostaining Western blotting	Cadmium	Wu et al. (2022)
2D	H1 and H9	24 well plate 12-day protocol	RT-qPCR	Halogenated flame retardants: TBBPA, TCBPA, TBBPS, TBBPA-BAE, TBBPA-BDBPE, and TBBPA-BHEE	Zhao et al. (2022)
	H9	Unspecified culture plate 12-day protocol	Cardiomyocyte contraction Cell proliferation during differentiation RT-qPCR RNAseq Western blotting Immunostaining	PFAS: F-35B and PFOS	Yang et al. (2020)
	H1 & H9 Mel1 NKX2.5-eGFP reporter cell line	Unspecified culture plate 17-day protocol	*NKX2.5*-eGFP reporter quantification RT-qPCR Immunostaining RNAseq CHIPseq Western blotting	TCDD	Fu et al. (2019)
	H9-MESP1-mTomato knock in	Unspecified culture plate 24-day protocol	Immunostaining Flow cytometry Proteomics	TiO2 nanoparticles	Pan et al. (2018)
	RUES2	Unspecified culture plate 15-day protocol	Cardiomyocyte contraction Immunostaining Flow cytometry	Extracts of cigarette smoke and e-cigarettes (mixtures)	Palpant et al. (2015)

*5-FU* 5-fluorouracil, *atRA* all-trans retinoic acid, *BPA* bisphenol A, *EBs* embryoid bodies, *F-35B* consists of 90% 6:2 chlorinated polyfluorinated ether sulfonate and 10% 8:2 chlorinated polyfluorinated ether sulfonate, *FBS* fetal bovine serum, GenX undecafluoro-2-methyl-3-oxahexanoic acid, hESCs human embryonic stem cells, *hiPSCs* human-induced pluripotent stem cells, *PFOA* perfluorooctanoic acid, *PFOS* Perfluorooctane sulfonic acid, RNAseq RNA sequencing, *RT-qPCR* reverse transcription quantitative PCR, *TBBPA* Tetrabromobisphenol A, *TBBPA-BAE* TBBPA-bis(allyl ether), *TBBPA-BDBPE* TBBPA-bis(23-dibromopropyl ether), *TBBPA-BHEE* TBBPA-bis(2-hydroxyethyl ether), *TBBPS* Tetrabromobisphenol S, *TCBPA* Tetrachlorobisphenol A, *TCDD* 2378-Tetrachlorodibenzodioxin, *VPA* valproic acid, *WT* wild type

### Developmental toxicity assays based on cardiomyocyte differentiation in human stem cells

It has been shown through 2D/3D comparison of stem cell differentiation that culturing 3D stem cell structures better resemble in vivo conditions than the adherent 2D cultures, as 3D cultures display higher functionality, increased long-term stability and ability to mimic features of whole organs (Fleischer et al. 2019; Zuppinger 2019; Zeevaert et al. 2020; Zink et al. 2020). On the other hand, Zhang et al. demonstrated in a basic research study that molecular mechanisms are highly comparable between 2D and 3D (Zhang et al. 2015). In a work of Wu et al., exposure to cadmium was shown to negatively affect cardiac maturation in both 2D and 3D models, though an effect at lower exposure levels was observed in the first model (Wu et al. 2022). This was attributed to a lower contact of the 3D organoids to the chemical when compared to the exposed monolayer, which also advocates for a more in vivo like effect of 3D organoids, though sacrificing the sensitivity of the assay.

There is an ethical distinction to be made between hESCs and hiPSCs that is related to the origin of the embryonic cell lines, where blastocysts are sacrificed (Zink et al. 2020). When comparing the response to chemical exposures, it has been demonstrated that hESCs and hiPSCs show comparable predictive value (Mayshar et al. 2011; Zhang et al. 2015; Liu et al. 2018). Thus, hiPSCs are a better choice from an ethical standpoint.

Ethical considerations are also relevant in relation to animal-derived supplements such as fetal bovine serum (FBS) that is added to the culture mediums and still used in some studies (Zhu et al. 2011; Aikawa et al. 2014; Fang et al. 2018; Cao et al. 2019; Aikawa 2020; Walker et al. 2021). In addition, the batch variations of non-defined medium supplements such as FBS introduce undesirable variability into the assay systems (Gstraunthaler et al. 2013).

From the summarized publications in Table 1, 11 studies are based on 2D adherent cell cultures, whereas 11 rely on 3D cultures and one study relies on utilizing both 2D and 3D. Although all these cell models rely on cardiomyocyte differentiation, there are some significant differences in assay designs. A look through the different studies reveals that the protocols for cardiac differentiation vary greatly in duration, ranging from 7 to 28 days. This has implications for the cardiomyocyte maturity, the duration of the chemical exposure, and therefore the costs of the assay, and the suitability for screening purposes.

Some systems are designed to maximize the descriptive output on mechanisms of action of toxicity during cardiomyocyte differentiation, whereas others are designed to prioritize speed, simplicity, and high throughput for screening purposes. The descriptiveness of these assays varies with some focusing on full scale -omics and spatial analysis (Liu et al. 2018; Pan et al. 2018; Fu et al. 2019; Yang et al. 2020; Lauschke et al. 2021a). Cardiomyocyte contraction is the original apical endpoint from the mEST and is also used in the majority of the publications.

Including the PluriLum assay, there are five human stem cell-based reporter cell systems for assessment of toxic effects reported. Three reporter systems are related to expression of *NKX2.5* (Fu et al. 2019; Lauschke et al. 2021b; Wu et al. 2022), one with *TNNT2* (Cao et al. 2019) and one with *MESP1* expression (Pan et al. 2018), and all of them, with the exception of the one developed by our group (Lauschke et al. 2021b), are based on hESCs.

### Assay responses to test compounds in PluriLum and PluriBeat, and relation to previous published data

#### 5-Fluorouracil and all-trans retinoic acid

The positive control, 5-FU, showed a toxic effect in both the PluriLum and the PluriBeat assay, exhibiting an effect in luminescence at 0.2 µM and at 3.2 µM on cardiomyocyte contractions.

5-FU is the best described compound in comparable assays. In a 3D hiPSC assay, an effect at 4.5 µM was reported (Aikawa 2020), while 2D systems reported an effect at 3 µM (Jamalpoor et al. 2022), at 1 µM (Galanjuk et al. 2022), and at 0.018 nM (Walker et al. 2021). When measuring reporter gene expression, slightly higher sensitivity is generally observed. For instance, an effect at 0.5 µM 5-FU was found in a 3D hEST-based assay by measuring the inhibition of the expression of the gene encoding for the alpha isoform of myosin heavy chain, a myocyte-specific protein involved in cardiac contraction and relaxation (Fang et al. 2018). Another reporter gene assay in 3D hiPSCs, in which reduction of *TNNT2* gene expression was used as the endpoint led to a 50% inhibition of differentiation (ID_50_) at 0.45 µM 5-FU (Cao et al. 2019).

This similar pattern across assays suggests that the assessment of gene expression may be a more sensitive endpoint than apical endpoints, such as cardiomyocyte contractions. Overall, a potent toxic effect of 5-FU is consistently observed across all assays.

To put the assay outputs into perspective, plasma levels of cancer patients undergoing treatment with 5-FU were found to range from nanomolar levels to as high as 13.8 µM (Beumer et al. 2009), indicating that stem cell-based assays provide sensitive outputs at relevant exposure levels.

Our second positive control, atRA, caused a potent effect in both the PluriBeat and the PluriLum assay with a LOAEL of 12.5 nM on beat score, and 200 nM in the PluriLum assay. Effect levels for this chemical on similar assays vary considerably. A LOAEL of 3.3 pM atRA has been reported for inhibition of cardiomyocyte beating in a 2D hiPSC model (Walker et al. 2021). On the other hand, an ID_50_ of 21 µM for the same endpoint was observed in a 3D hiPSCs-based assay (Aikawa 2020). Another study using a 3D hiPSC-based cardiomyocyte differentiation assay reported a LOAEL for beating of 100 nM and a complete lack of differentiation at 10 µM (Hoang et al. 2021). This latter finding is more closely aligned with the sensitivity shown by our models.

atRA is a well-known developmental toxicant in humans and animals (Piersma et al. 2017) and human teratogenicity has previously been associated with vitamin A supplement intake during pregnancy, leading to a wide range of birth defects (Rothman et al. 1996). Normal non-teratogenic blood serum levels have been reported to be around 5 nM in pregnant women (Czuba et al. 2022).

A large variation in sensitivity to atRA is observed in literature, but the PluriBeat shows a sensitivity close to biologically relevant levels. When comparing the tendency of the effects of atRA in the PluriBeat and the PluriLum assays, we can see an indication of a similar trend of effect. This trend could suggest that the effect in the PluriLum assay is consistent with that of the PluriBeat, but that large variation between experiments in the PluriLum assay has resulted in statistically non-significant results. Replication of the experiments would be able to elucidate this question.

#### Valproic acid, folic acid and ascorbic acid

We observed no statistically significant effect of VPA on beating nor on *NKX2.5*-derived luminescence at an exposure range up to 300 µM, although a tendency towards an inhibition of *NKX2.5* expression was seen at the highest test concentration. Our results are in line with our previous observations (Lauschke et al. 2020, 2021b). In a 2D hiPSC-based assay, VPA exposure affected cardiomyocyte beating with a LOAEL of 196 µM (Jamalpoor et al. 2022), while in a 3D hiPSC model a beat-derived ID_50_ of 868 µM is reported in 2014 (Aikawa et al. 2014), while it was later reported a beat-derived ID_50_ of 67 µM in 2020 in the same test system (Aikawa 2020). Overall, VPA shows either weak or no effects on cardiomyocyte contractions.

For comparison, human exposure levels of VPA of up to 305 µM in cord blood have been reported (Koch et al. 1996), illustrating the relevance of stem cell-based models for the assessment of embryotoxic effects of VPA.

VPA has been associated with human teratogenicity leading to increased incidents of for instance neural tube defects and cleft palate (Jentink et al. 2010). The inability of the PluriLum and PluriBeat assay to detect effects of VPA highlights a limitation of the assays. Though effects on expression of *NKX2.5* have been reported (de Jong et al. 2011), assays relying on differentiation towards neuronal lineages or all three germ layers appear to be more sensitive to developmental effects of VPA (Shinde et al. 2016, 2017; Konala et al. 2021), suggesting that assays based on cardiomyocyte differentiation might not be suitable for detecting compounds exerting effects on other germ layers including neural development.

As expected, for the negative control folic acid, we observed no effect in the PluriLum assay. However, there were statistically significant effects on the beat score at concentrations of 25 and 100 µM, but not at 50 µM. The lack of effect in the generally more sensitive PluriLum assay suggests that the inconsistent effects observed in the PluriBeat are most likely false positives. For comparison, limited data is available besides the work by Jamalpoor et al., in which no effect was observed on beat score in a 2D hiPSCs assay following exposure to 1.9 µM folic acid (Jamalpoor et al. 2022). Thus, by using the objective luminescent endpoint of the PluriLum assay, we might eliminate false positives that could stem from subjective assessments of effects in the PluriBeat assay.

Also assessed as a known negative control, we observed no effect of ascorbic acid tested up to 500 µM in neither the PluriLum nor the PluriBeat assay. Previously, a beat score reduction was reported in a 3D hiPSC model with an ID_50_ of 17 mM ascorbic acid (Aikawa 2020). However, the concentration range tested in that study does not bear any biological relevance.

#### Perfluoroalkyl substances

When comparing effects of PFAS in both assays in the present work, PluriLum displayed higher sensitivity, being able to identify embryotoxicity for all three chemicals with a LOAEL for PFOS, PFOA, and GenX of 12.5, 6.3 and 50 µM. In the PluriBeat assay, PFOS gave rise to a significant decline in the number of beating EBs, while GenX led to no response, which is in line with previous findings from the parent cell line of the cell line used in this work (Davidsen et al. 2021). In contrast to earlier findings (Davidsen et al. 2021), PFOA did not significantly affect beat score in this study, which might stem from interpersonal handling differences and/or the subjectiveness of beat scoring.

PFOS has been tested in a 2D hESC cardiomyocyte developmental toxicity assay reporting a LOAEL on cardiomyocyte beating at 60 µM (Yang et al. 2020), a level which is similar to results from the mEST, where an effect at 73 µM (ID_50_) was reported (Zhou et al. 2017). In a previous work from our group using the PluriBeat assay, a LOAEL of 6.3 µM PFOS was observed in EBs (Davidsen et al. 2021), which is comparable to the sensitivity found in this study (12.5 µM) in both the PluriLum and the PluriBeat assay.

No data was found for PFOA on human-based cardiomyocyte differentiation in vitro, but an effect at 213 µM (ID_50_) has been reported in the mEST (Zhou et al. 2017). The PluriLum assay picked up an effect at 6.3 µM, indicating an improved sensitivity compared to the mEST.

We also observed an effect of GenX in the PluriLum assay with a LOAEL of 50 µM. This effect is close to the reported effect from 25 µM by Davidsen in the cell line IMR90-1 (Davidsen et al. 2021). GenX has been reported to cause an increased heart rate at 6 µM in the Zebrafish embryo assay (Gong et al. 2023).

Generally, human serum levels of PFOS and PFOA are lower than the LOAELs in this study. However, occupational exposure can lead to extremely high blood levels of PFAS, as reported in Chinese chemical plant workers, who had serum levels as high as 236 µM PFOS and 77 µM PFOA (Fu et al. 2016), substantiating the relevance of the PluriLum findings for highly exposed individuals. Limited human exposure levels of GenX are available, with a single report on the prevalence of GenX in 24 American serum samples showing levels in the range of 0.003–0.15 μM (Robarts et al. 2022).

Importantly, both PFOA and PFOS exposure during pregnancy have been associated with reduced birth weight in humans (Maisonet et al. 2012; Lenters et al. 2016; Schrenk et al. 2020), and our results suggest that the PluriLum assay could be a valuable tool for the study of the mechanisms underlying this known embryotoxicity, as well as for the screening of PFAS in general for embryotoxic effects.

#### Conazole fungicides

Both epoxiconazole and prochloraz were observed to reduce luminescence intensity in the PluriLum assay, with LOAELs of 5 µM and 2.5 µM, respectively. Epoxiconazole had a non-significant impact on the beat score, whereas prochloraz did not cause any effect on cardiomyocyte beating. This effect of epoxiconazole is in line with our previous findings, in which a LOAEL of 2.5 µM on beat score was reported (Lauschke et al. 2020).

Only limited data from similar in vitro sources on these fungicides are available for comparison. However, both compounds have been tested in the mEST, where they were shown to inhibit 3D cardiomyocyte differentiation in murine D3 cells with an ID_50_ of 34 µM for epoxiconazole and an ID_50_ of 37 µM for prochloraz (Dreisig et al., 2013).

Both epoxiconazole and prochloraz are known developmental toxicants in rat studies, where they affect sex hormone levels, cause malformations among several other adverse effects (Dreisig et al., 2013). Moreover, we have previously observed that epoxiconazole is affecting cholesterol synthesis and steroidogenesis in the PluriBeat assay (Lauschke et al. 2021a, b) and thus, it is possible that these conazole fungicides affect cardiomyocyte differentiation via adverse effects on precursors of steroidogenesis.

## Conclusion

Compared to the existing human stem cell-based assays measuring cardiomyocyte differentiation, we conclude that our findings in this study generally align with the reported outcomes in the literature. All in all, we observed that the Plurilum assay appears to detain higher sensitivity in picking up the developmental toxic potential of various classes of chemicals, in some cases detecting an effect that was absent from the original assessment of cardiomyocyte beating. Thus, our work demonstrates that the use of the reporter gene assay PluriLum may lead to a reduction in the number of false negatives which might be observed when using beat score as the output, as it was the case for both conazole fungicides, PFOA and GenX, where an effect was observed in the PluriLum, but not in the PluriBeat assay. Moreover, the number of false positives may be reduced as well, as shown for folic acid.

The absence of effect of VPA in the PluriLum assay emphasizes one of the limitations of this assay as it may not be able to detect toxicants causing developmental neurotoxicity. Importantly, one single assay based on cardiomyocyte differentiation does not express all relevant signaling pathways and cellular processes in the developing embryo/fetus, which highlights that the assay cannot stand alone and that a panel of tests is needed for a thorough developmental toxicity assessment.

Further optimizations of the PluriLum assay might enhance its descriptive ability — improvements could include standardization of spheroid formation and differentiation, alleviating some of the variability between experimental runs. Nevertheless, it seems that several chemicals exert effects in this assay at test concentrations which can be close to human exposure levels.

The field of in vitro toxicology is on the verge of gaining regulatory acceptance. Assays for developmental toxicity testing based on human stem cell cultures and cardiomyocyte differentiation have the potential to reduce the cost and lead time of current testing regimes, making it possible to lower the barrier for what is worth testing in both pharmaceutical industries and basic science. With an increasing demand for toxicity testing, a method like the PluriLum assay can contribute to the in vitro toolbox for describing chemicals with potential harmful developmental outcomes in humans.

## Data Availability

The datasets generated during and/or analyzed during the current study are available from the corresponding author on reasonable request.

## Footnotes

1 https://cran.r-project.org/web/packages/MASS/index.html.
